# The clinical usage of liposomal amphotericin B in patients receiving renal replacement therapy in Japan: a nationwide observational study

**DOI:** 10.1007/s10157-020-01989-3

**Published:** 2020-11-11

**Authors:** Yoko Obata, Takahiro Takazono, Masato Tashiro, Yuki Ota, Tomotaro Wakamura, Akinori Takahashi, Kumiko Sato, Taiga Miyazaki, Tomoya Nishino, Koichi Izumikawa

**Affiliations:** 1 Department of Nephrology, Nagasaki University Hospital, 1-7-1 Sakamoto, Nagasaki, 852-8501 Japan; 2 Department of Infectious Diseases, Nagasaki University Graduate School of Biomedical Sciences, 1-7-1 Sakamoto, Nagasaki, 852-8501 Japan; 3 Department of Respiratory Medicine, Nagasaki University Hospital, 1-7-1 Sakamoto, Nagasaki, 852-8501 Japan; 4 Nagasaki University Infection Control and Education Center, Nagasaki University Hospital, 1-7-1 Sakamoto, Nagasaki, 852-8501 Japan; 5 Medical Affairs Division, Sumitomo Dainippon Pharma Co., Ltd, 1-13-1 Kyobashi, Chuo-ku, Tokyo, 104-8356 Japan; 6 Deloitte Tohmatsu Consulting LLC, Marunouchi Nijubashi Building, 3-2-3 Marunouchi, Chiyoda-ku, Tokyo, 100-8361 Japan

**Keywords:** Liposomal amphotericin B, Maintenance hemodialysis, Continuous renal replacement therapy, Renal replacement therapy, Renal dysfunction

## Abstract

**Background:**

Liposomal amphotericin B (L-AMB), a broad-spectrum antifungicidal drug, is often used to treat fungal infections. However, clinical evidence of its use in patients with renal dysfunction, especially those receiving renal replacement therapy (RRT), is limited. Therefore, we evaluated the usage and occurrence of adverse reactions during L-AMB therapy in patients undergoing RRT.

**Methods:**

Using claims data and laboratory data, we retrospectively evaluated patients who were administered L-AMB. The presence of comorbidities, mortality rate, treatment with L-AMB and other anti-infective agents, and the incidence of adverse reactions were compared between patients receiving RRT, including continuous renal replacement therapy (CRRT) and maintenance hemodialysis (HD), and those that did not receive RRT.

**Results:**

In total, 900 cases met the eligibility criteria: 24, 19, and 842 cases in the maintenance HD, CRRT, and non-RRT groups, respectively. Of the patients administered L-AMB, mortality at discharge was higher for those undergoing either CRRT (15/19; 79%) or maintenance HD (16/24; 67%) than for those not receiving RRT (353/842; 42%). After propensity score matching, the average daily and cumulative dose, treatment duration, and dosing interval for L-AMB were not significantly different between patients receiving and not receiving RRT. L-AMB was used as the first-line antifungal agent for patients undergoing CRRT in most cases (12/19; 63%). Although the number of subjects was limited, the incidence of adverse events did not markedly differ among the groups.

**Conclusion:**

L-AMB may be used for patients undergoing maintenance HD or CRRT without any dosing, duration, or interval adjustments.

## Introduction

Invasive fungal infections frequently occur in immunocompromised and critically ill patients and are associated with high morbidity and mortality [1–5]. Amphotericin B is a broad-spectrum antifungicidal drug that is used against most clinically relevant yeasts and molds that cause mycoses such as aspergillosis, candidiasis, cryptococcosis, and mucormycosis [6]. However, the use of amphotericin B has been limited because of its high incidence of toxicities such as nephrotoxicity, liver disorder, hypokalemia, or fever [6, 7]. Liposomal amphotericin B (L-AMB), which encapsulates amphotericin B within a liposomal membrane, was developed to reduce toxicity while maintaining the antifungal activity of amphotericin B [8, 9]. The safety and efficacy of L-AMB were found to be improved in patients with impaired renal function [10, 11] or in other high-risk patients [12, 13]. Reduced renal dysfunction in patients receiving L-AMB is supported by animal studies that reveal the predominant involvement of the liver in L-AMB clearance and the limited contribution of the kidneys [14]. L-AMB accumulates in the liver and spleen, rather than in the kidneys; thus, only 4.5% of the drug is excreted in urine in an unchanged form [6, 15]. Despite its reduced nephrotoxicity, physicians are reluctant to prescribe L-AMB, as there is limited clinical evidence to support its use in patients with renal dysfunction, especially those receiving renal replacement therapy (RRT).

Several studies from single facilities have reported that in critically ill patients receiving RRT, adjustment of the L-AMB dose is not necessary for those undergoing maintenance hemodialysis (HD) or continuous renal replacement therapy (CRRT). This is because RRT may not affect the efficacy and pharmacokinetics of L-AMB [16–20]. Studies conducted in Japan and other regions of the world have revealed that L-AMB is equally effective in patients receiving RRT, including maintenance HD or CRRT, as it is in those who do not receive RRT therapy, despite the limited number of study participants [16, 17]. In addition, in patients undergoing CRRT treated with L-AMB, no significant differences were observed in the L-AMB pharmacokinetic parameters compared with other patients in the group [18]. Another case study revealed no decline in the serum concentration of amphotericin B during L-AMB treatment in patients receiving HD [19, 20]. However, a large-scale study using multicenter data is needed to verify these findings. Therefore, the daily practice of L-AMB treatment in patients receiving RRT, especially maintenance HD and CRRT, must be examined to ensure that it is appropriately administered to patients.

In this study, we employed claims data to investigate the clinical usage of L-AMB in patients with renal dysfunction undergoing maintenance HD and CRRT in Japan. We also evaluated the comorbidities, mortality, treatment with L-AMB and other anti-infective (antifungal and antimicrobial) agents, and the incidence of adverse events for these patients.

## Materials and methods

### Data source

This retrospective, multicenter, observational study was based on the data retrieved between April 2008 and January 2018 from an electronic medical information database (Medical Data Vision Co., Ltd.). This database contains diagnosis procedure combination (DPC) hospital data, medical fee reimbursement claims, and clinical laboratory test data from 345 facilities in Japan. Baseline patient information included age, sex, diagnosis, and comorbidities at admission, coded using the International Classification of Diseases, 10th Revision (ICD-10) codes. The database also contained all drug dosages and administration dates during hospitalization. All interventional procedures were decoded from the original Japanese codes. All subjects in this study were admitted to public, private, or government hospitals, but not university hospitals, and all hospitals had 200 or more beds.

### Study design

Patients administered L-AMB during hospitalization were included in the study. Patients with an L-AMB administration interval of ≥ 8 days were categorized as multiple cases. To identify study subjects, patients aged < 18 years on the first day of the month of L-AMB administration and those administered average daily L-AMB doses of > 6 mg/kg body weight were excluded. Patients were also excluded when body weight was not available to calculate the administered dose of L-AMB. RRT was defined as a procedure for artificial kidneys, CRRT, plasma exchange, hemoadsorption, cytapheresis, peritoneal dialysis, and regional perfusion and was performed on the day of and within 3 days before the initiation of L-AMB administration. RRT was classified into maintenance HD and CRRT. Maintenance HD was defined as a procedure for artificial kidneys [Japanese Procedure Code: J0381, J0382, and J0383] or a procedure involving the use of a dialyzer/hollow fiber/HD filter on the day of and within 7 days before the initiation of L-AMB administration. Procedures defined by J0382 were not identified in the study. Patients must have been treated within three consecutive days at an interval of ≤ 2 days to be assigned to the maintenance HD group. CRRT was defined as the procedure performed for CRRT (Japanese Procedure Code: J038-2) only on the day of L-AMB treatment initiation. Patients undergoing simultaneous procedures for plasma exchange/hemoadsorption/hollow fiber/HD filters were included in the CRRT group. Patients who did not fit into any of the two categories and those who underwent both procedures were not evaluated in this study. A non-RRT patient was defined as a subject that did not receive RRT.

### Assessments

The duration of L-AMB therapy was defined as the time from treatment initiation to treatment discontinuation (drug interval: ≥ 8 days). To calculate the dosing interval, patients who received single L-AMB administration were excluded; one interval signified continuous administration. The first-line use of L-AMB was defined as no treatment with antifungal agents on the day of and within 7 days before the initiation of L-AMB administration. The antibacterial and antifungal drugs administered prior to L-AMB therapy were identified on the day of and within 7 days before the initiation of L-AMB administration. Concomitant antibacterial and antifungal drugs were identified from the day after the initiation of L-AMB administration to the day before the end of treatment. Patients who were administered L-AMB for 1 or 2 days were excluded from the denominator. The antibacterial and antifungal drugs administered following L-AMB therapy were identified between the final day of L-AMB treatment and 7 days after treatment termination. Comorbidities were identified from the month of L-AMB therapy initiation to the final month of therapy. Common Terminology Criteria for Adverse Events (CTCAE) grade ≥ 3 standards except for death were used to define adverse drug reactions according to clinical laboratory tests, which were performed between the day following L-AMB treatment initiation and 7 days after treatment termination. Patients within the range of the CTCAE standard on the day of and within 7 days before L-AMB treatment initiation were subjected to analysis. Rhabdomyolysis was defined using the ICD10 classification code M6289, and anaphylaxis was defined using the ICD10 codes T780 and T782 from the month of L-AMB therapy initiation to the final month of therapy. Countershock was defined using the Japanese Procedure Code J0471/J0472, and internal cardiac massage was defined using the Japanese Procedure Code K545 between the day following L-AMB treatment initiation and 7 days after treatment termination.

### Statistical analysis

Propensity scores were calculated using a logistic regression model and the following covariates: age; sex; the presence of comorbidities: septic shock and disseminated intravascular coagulation; and catecholamine treatment between the day before the initiation of L-AMB treatment and 7 days after treatment termination. Using these propensity scores, maintenance HD and CRRT cases were individually matched with non-RRT cases at a 1-to-1 ratio using the nearest matching method within 0.1 caliper distance. If multiple cases with the same propensity score were matched, a case with the nearest start date of L-AMB treatment was selected. After matching, a paired student’s *t* test was performed to compare the average of daily and cumulative dose, duration, and dosing interval of L-AMB treatment. To evaluate the differences between patients undergoing either maintenance HD or CRRT and patients not receiving RRT, Welch’s *t* test was performed to compare the average age, and the Fisher’s exact test was performed to compare the categorical variables. Because all analyses were performed in an exploratory manner, no adjustment for multiplicity was performed.

## Results

### Study population and patient characteristics

**Fig. 1:**
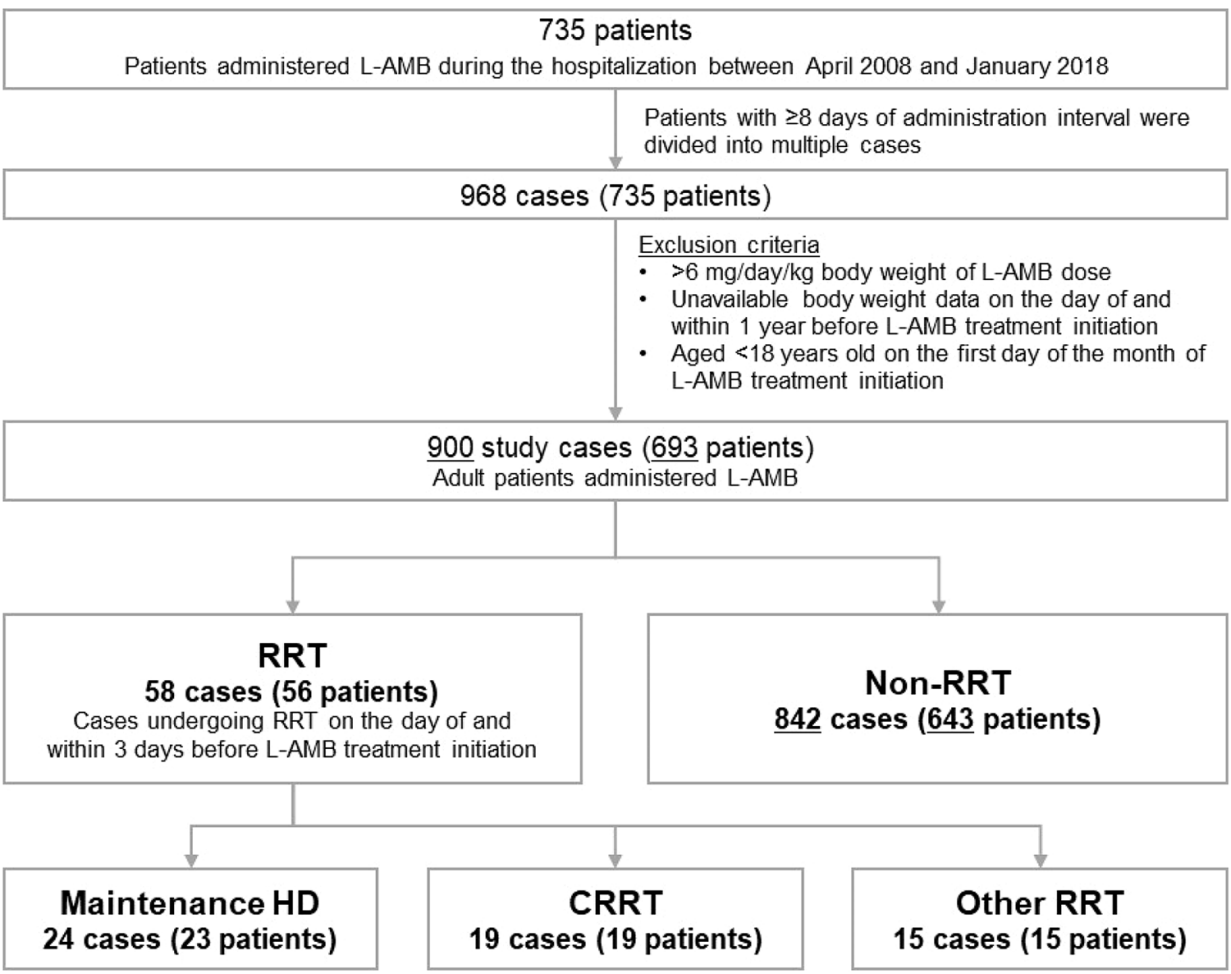
Study population. *L-AMB* Liposomal amphotericin B, *RRT* renal replacement therapy, *HD* hemodialysis, *CRRT* continuous renal replacement therapy

A total of 900 cases met the eligibility criteria and were included in the study (Fig. 1). Twenty-four cases in the maintenance HD group, 19 cases in the CRRT group, and 842 in the non-RRT group were analyzed, whereas 15 other RRT cases were not evaluated. The characteristics of patients are presented in Table 1. The proportion of male patients was higher in the CRRT group than in the non-RRT group; however, the average age was similar among the groups. Mortality at hospital discharge was significantly higher in the maintenance HD (16/24; 67%) and CRRT (15/19; 79%) groups than in the non-RRT group (353/842; 42%). Overall, 81% (13/16) of patients in the maintenance HD group died within 7 days after the completion of L-AMB treatment. Additionally, 73% (11/15) of patients in the CRRT group died within the day following L-AMB treatment completion, and 10 of the deceased patients received short-term L-AMB treatment within 3 days. A significantly higher incidence of septic shock was observed in the deceased patients in the maintenance HD group (5/16; 31%) and the CRRT group (9/15; 60%) than those in the non-RRT group (29/353; 8%). The incidence of disseminated intravascular coagulation was significantly higher in the CRRT group (12/15; 80%) than in the non-RRT group (90/353; 25%). At the start date of L-AMB treatment, most patients undergoing maintenance HD were treated in the Hematology, Internal, and Nephrology departments, whereas those undergoing CRRT were treated in the Hematology, Internal, and Surgery departments. The majority of patients in the non-RRT group (522/842; 62%) were treated in the Hematology department.

**Table 1:** Characteristics of patients undergoing maintenance HD and CRRT and patients who did not receive RRT

Patient characteristics	Maintenance HD (*N* = 24)	CRRT (*N* = 19)	Non-RRT (*N* = 842)
Sex, male (%)	18 (75%, *P* = 0.511)	**17 (89%, P = 0.046)**	557 (66%)
Age (mean ± standard deviation)	65.1 ± 12.3 (*P* = 0.648)	65.5 ± 14.0 (*P* = 0.636)	63.9 ± 16.1
≧ 65 years	14 (58%, *P* = 1.000)	11 (58%, *P* = 1.000)	479 (57%)
Mortality (%)	**16 (67%, P = 0.020)**	**15 (79%, P = 0.002)**	353 (42%)
Pattern of mortality	(*N* = 16)	(*N* = 15)	(*N* = 353)
Within 7 days after the termination of L-AMB treatment	**13 (81%, P = 0.037)**	12 (80%, *P* = 0.061)	186 (53%)
Within the following day after termination of L-AMB treatment	7 (44%, *P* = 0.610)	**11 (73%, P = 0.012)**	133 (38%)
Of above, cases with L-AMB treatment for 3 days or shorter	0 (0%, *P* = 0.382)	**10 (67%, P < 0.001)**	35 (10%)
After 8 days or later after the termination of L-AMB treatment	**3 (19%, P = 0.037)**	3 (20%, *P* = 0.061)	167 (47%)
Comorbidities in mortality cases	(*N* = 16)	(*N* = 15)	(*N* = 353)
Septic shock	**5 (31%, P = 0.010**)	**9 (60%, P < 0.001**)	29 (8%)
Disseminated intravascular coagulation	5 (31%, *P* = 0.569)	**12 (80%, P < 0.001**)	90 (25%)
Treatment department (%)			
Hematology	**6 (25%, P < 0.001)**	**4 (21%, P < 0.001)**	522 (62%)
Internal	6 (25%, *P* = 0.424)	4 (21%, *P* = 0.765)	155 (18%)
Hematologic Oncology	0 (0%, *P* = 0.622)	0 (0%, *P* = 1.000)	40 (5%)
Rehabilitation	2 (8%, *P* = 0.305)	0 (0%, *P* = 1.000)	38 (5%)
Surgical	1 (4%, *P* = 1.000)	**3 (16%, P = 0.041)**	33 (4%)
Nephrology	**5 (21%, P < 0.001)**	0 (0%, *P* = 1.000)	8 (1%)
Diagnosis (%)			
Aspergillosis	3 (13%, *P* = 0.108)	**1 (5%, P = 0.034)**	236 (28%)
Candidiasis	1 (4%, *P* = 1.000)	0 (0%, *P* = 0.632)	58 (7%)
Cryptococcosis	0 (0%, *P* = 1.000)	0 (0%, *P* = 1.000)	15 (2%)
Zygomycosis	0 (0%, *P* = 1.000)	0 (0%, *P* = 1.000)	11 (1%)
Aspergillosis and candidiasis	0 (0%, *P* = 1.000)	0 (0%, *P* = 1.000)	2 (0.2%)
Aspergillosis and cryptococcosis	0 (0%, *P* = 1.000)	0 (0%, *P* = 1.000)	1 (0.1%)
Aspergillosis, candidiasis, and cryptococcosis	0 (0%, *P* = 1.000)	0 (0%, *P* = 1.000)	1 (0.1%)
Other fungal infections	9 (38%, *P* = 0.511)	3 (16%, *P* = 0.210)	264 (31%)
Unknown	11 (46%, *P* = 0.116)	**15 (79%, P < 0.001)**	254 (30%)
Unknown except neutropenia	**11 (46%, P = 0.010)**	**14 (74%, P < 0.001)**	181 (21%)
Neutropenia	0 (0%, *P* = 0.253)	1 (5%, *P* = 1.000)	73 (9%)

Bold values indicate statistically significant *P* values (*P* < 0.05)

*N* represents the number of cases. Welch’s *t* test for continuous variables or the Fisher’s exact test for categorical variables was performed to compare the maintenance HD group or the CRRT group to the non-RRT group. Other fungal infections included coccidioidomycosis, blastomycosis, maduramycosis, and unclassified or unspecified mycosis. Bold font indicates *P* < 0.05

*L-AMB* Liposomal amphotericin B, *RRT* renal replacement therapy, *HD* hemodialysis, *CRRT* continuous renal replacement therapy

### L-AMB and anti-infective agent treatment

In all groups, antibiotic agents were administered prior, concomitantly, and after L-AMB administration. There was no significant difference in the frequency of concomitant antibiotic agent use (Table 2). Carbapenem and anti-MRSA drugs were commonly used among the groups (Table 2).

**Table 2:** Antibiotic treatment in patients undergoing maintenance HD and CRRT and patients who did not receive RRT

Treatment	Maintenance HD (*N* = 24)	CRRT (*N* = 19)	Non-RRT (*N* = 842)
Antibiotic treatment (%)			
Prior to L-AMB treatment	23 (96%, *P* = 1.000)	19 (100%, *P* = 0.389)	779 (93%)
Concomitant treatment	23 (100%, *N* = 23, *P* = 0.248)	11 (100%, *N* = 11, *P* = 0.613)	708 (91%, *N* = 774)
Following L-AMB treatment	21 (88%, *P* = 0.783)	19 (100%, *P* = 0.056)	703 (83%)
Concomitant antibiotics (%)	(*N* = 23)	(*N* = 11)	(*N* = 708)
Penicillin	6 (26%, *P* = 1.000)	2 (18%, *P* = 0.736)	198 (28%)
Cephem	7 (30%, *P* = 0.636)	2 (18%, *P* = 0.737)	186 (26%)
Carbapenem	9 (39%, *P* = 0.133)	8 (73%, *P* = 0.367)	402 (57%)
Aminoglycoside	2 (9%, *P* = 0.757)	4 (36%, *P* = 0.057)	98 (14%)
Quinolone	10 (43%, *P* = 0.251)	2 (18%, *P* = 0.518)	217 (31%)
Trimethoprim	8 (35%, *P* = 1.000)	2 (18%, *P* = 0.228)	264 (37%)
Anti-MRSA drug	9 (39%, *P* = 0.665)	5 (45%, *P* = 0.531)	249 (35%)
Other antibiotics	9 (39%, *P* = 0.359)	3 (27%, *P* = 1.000)	211 (30%)

*N* represents the number of cases. Fisher’s exact test was performed to compare the maintenance HD group or the CRRT group with the non-RRT group

*L-AMB* Liposomal amphotericin B, *RRT* renal replacement therapy, *HD* hemodialysis, *CRRT* continuous renal replacement therapy, *MRSA* methicillin-resistant Staphylococcus aureus

To balance the severity of illness and patient characteristics, propensity score matching was performed according to a multiple logistic regression model. Following propensity score matching, there was no significant difference in the average daily and cumulative administered dose of L-AMB between the maintenance HD (2.50 ± 0.77 mg/kg/day and 45.27 ± 43.81 mg/kg) and the non-RRT groups (2.58 ± 0.80 mg/kg/day and 29.30 ± 24.47 mg/kg) or between the CRRT (2.56 ± 0.64 mg/kg/day and 18.70 ± 20.41 mg/kg) and the non-RRT groups (2.37 ± 0.96 mg/kg/day and 18.81 ± 13.49 mg/kg) (Table 3). In addition, the duration of L-AMB therapy in the maintenance HD or CRRT group was not significantly different from that in the non-RRT group (Table 3). Patients in the maintenance HD, CRRT, and non-RRT groups received continuous L-AMB treatment (Table 3).

**Table 3:** L-AMB administration in patients undergoing maintenance HD and CRRT and patients who did not receive RRT after propensity score matching

L-AMB administration	Maintenance HD	Non-RRT for maintenance HD	CRRT	Non-RRT for CRRT
Average daily dose (mg/kg/day)	2.50 ± 0.77 (*N* = 22, *P* = 0.739)	2.58 ± 0.80 (*N* = 22)	2.56 ± 0.64 (*N* = 14, *P* = 0.559)	2.37 ± 0.96 (*N* = 14)
Cumulative dose (mg/kg)	45.27 ± 43.81 (*N* = 22, *P* = 0.155)	29.30 ± 24.47 (*N* = 22)	18.70 ± 20.41 (*N* = 14, *P* = 0.985)	18.81 ± 13.49 (*N* = 14)
Duration (day)	18.5 ± 17.9 (*N* = 22, *P* = 0.118)	11.1 ± 8.8 (*N* = 22)	8.4 ± 13.0 (*N* = 14, *P* = 0.891)	8.9 ± 6.1 (*N* = 14)
Dosing interval (day)	1.0 ± 0.0 (*N* = 20, *P* = 0.190)	1.0 ± 0.1 (*N* = 20)	1.0 ± 0.0 (*N* = 12, *P* = 0.178)	1.1 ± 0.2 (*N* = 12)

Bold values indicate statistically significant *P* values (*P* < 0.05)

*N* represents the number of cases. Data are expressed as mean ± standard deviation. A paired student’s *t* test was performed to compare the maintenance HD group or the CRRT group to the non-RRT group

*L-AMB* Liposomal amphotericin B, *RRT* renal replacement therapy, *HD* hemodialysis, *CRRT* continuous renal replacement therapy

L-AMB was significantly more frequently used as the first-line treatment in the CRRT (12/19; 63%) than in the non-RRT groups (235/842; 28%) (*P* = 0.002), whereas a similar frequency was observed between the maintenance HD (7/24; 29%) and the non-RRT groups (Table 4). For patients administered L-AMB as the second and/or later-line treatment, micafungin, caspofungin, and fluconazole were commonly administered prior to L-AMB treatment in all groups (Table 4). Following the completion of L-AMB treatment, although over half of the patients in the non-RRT group (486/842; 58%) received antifungal agents, except L-AMB, this frequency was lower in the maintenance HD (7/24; 29%) and the CRRT groups (3/19; 16%) (Table 4).

**Table 4:** Antifungal treatment in patients undergoing maintenance HD and CRRT and patients who did not receive RRT

Treatment	Maintenance HD (*N* = 24)	CRRT (*N* = 19)	Non-RRT (*N* = 842)
Antifungal drugs			
L-AMB as the first-line drug	7 (29%, *P* = 0.822)	**12 (63%, P = 0.002)**	235 (28%)
Other antifungal drugs			
Prior to L-AMB treatment	17 (71%, *P* = 0.822)	**7 (37%, P = 0.002)**	607 (72%)
Concomitant treatment	**1 (4%, N = 23, P = 0.004)**	1 (9%, *N* = 11, *P* = 0.187)	241 (31%, *N* = 774)
Following L-AMB treatment	**7 (29%, P = 0.006)**	**3 (16%, P < 0.001)**	486 (58%)
Antifungal drugs administered prior to L-AMB	(*N* = 17)	(*N* = 7)	(*N* = 607)
Azoles			
Fluconazole	2 (12%, *P* = 0.388)	3 (43%, *P* = 0.192)	135 (22%)
Itraconazole	1 (6%, *P* = 0.709)	0 (0%, *P* = 1.000)	68 (11%)
Voriconazole	2 (12%, *P* = 0.545)	0 (0%, *P* = 0.355)	124 (20%)
Echinocandins			
Micafungin	11 (65%, *P* = 0.139)	4 (57%, *P* = 0.707)	274 (45%)
Caspofungin	2 (12%, *P* = 0.385)	2 (29%, *P* = 0.665)	140 (23%)
5-Fluoropyrimidine			
Flucytosine	0 (0%, *P* = 1.000)	0 (0%, *P* = 1.000)	13 (2%)

Bold values indicate statistically significant *P* values (*P* < 0.05)

*N* represents the number of cases. Fisher’s exact test was performed to compare the maintenance HD group or the CRRT group with the non-RRT group. Bold font indicates P < 0.05

*L-AMB* Liposomal amphotericin B, *RRT* renal replacement therapy, *HD* hemodialysis, *CRRT* continuous renal replacement therapy

### Incidence of adverse drug reactions in patients receiving L-AMB

The incidence of adverse drug reactions such as hyperbilirubinemia, hypokalemia, thrombocytopenia, leukopenia, hepatic disorder, rhabdomyolysis, anaphylaxis, and cardiac arrest during L-AMB therapy was identified between the day after treatment initiation and 7 days after treatment completion. No significant difference was observed among the groups, albeit the limited number of subjects assessed (Table 5).

**Table 5:** Incidence of adverse drug reactions in patients undergoing maintenance HD and CRRT and patients who did not receive RRT

Disease	Definition	Maintenance HD (*N* = 24)	CRRT (*N* = 19)	Non-RRT (*N* = 842)
Hyperbilirubinemia/jaundice	Total bilirubin (> 4.5 mg/dL)	2/14 (14%, *P* = 0.092)	0/5 (0%, *P* = 1.000)	17/496 (3%)
Hypokalemia	Potassium (< 3 mEq/L)	1/12 (8%, *P* = 0.194)	1/12 (8%, *P* = 0.194)	144/510 (28%)
Thrombocytopenia	Thrombocyte (< 50,000/dL)	1/7 (14%, *P* = 0.570)	0/1 (0%, *P* = 1.000)	15/137 (11%)
Leukopenia	Leukocyte (< 2,000/dL)	0/10 (0%, *P* = 0.122)	0/1 (0%, *P* = 1.000)	47/201 (23%)
Hepatic disorder	AST (> 150 U/L)	1/12 (8%, *P* = 0.504)	0/4 (0%, *P* = 1.000)	18/328 (5%)
	ALT (male: > 210 U/L, female: > 115 U/L)	0/14 (0%, *P* = 0.613)	0/6 (0%, *P* = 1.000)	27/396 (7%)
Rhabdomyolysis	CK (male: > 1240 IU/L, female: > 765 IU/L)	0/6 (0%, *P* = 1.000)	0/5 (0%, *P* = 1.000)	2/54 (4%)
	CK abnormality + ICD10 code: M6289	0/6 (0%, *P* = 1.000)	0/5 (0%, *P* = 1.000)	0/54 (0%)
Anaphylaxis	ICD10 code: T780/T782	0/24 (0%, *P* = 1.000)	0/19 (0%, *P* = 1.000)	3/842 (0.4%)
Cardiac arrest	Counter-shock (ICD10 code: J0471/J0472) or open cardiac massage (ICD10 code: K545)	0/24 (0%, *P* = 1.000)	1/19 (5%, *P* = 0.106)	4/842 (0.5%)

*N* represents the number of cases. The number of subjects against each disease is shown in the denominator. Fisher’s exact test was performed to compare the maintenance HD group or the CRRT group to the non-RRT group

*L-AMB* Liposomal amphotericin B, *RRT* renal replacement therapy, *HD* hemodialysis, *CRRT* continuous renal replacement therapy, *ICD10* International Classification of Diseases 10th Revision, *AST* aspartate aminotransferase, *ALT* alanine transaminase, *CK* creatine kinase

## Discussion

Although the dose of renal excretory antibiotics such as carbapenem and azole antifungal agents must be adjusted before they are administered to patients receiving RRT [21, 22], several studies from single facilities have reported that the same dose of L-AMB could be administered to patients who either received or did not receive RRT. This is because RRT such as maintenance HD and CRRT does not affect the efficacy and pharmacokinetics of L-AMB [16–20]. However, large-scale clinical evidence from multiple facilities on the use of L-AMB in patients receiving RRT is needed to ensure that patients are appropriately administered this agent. Based on the claims data for patients administered L-AMB, we compared the clinical usage of L-AMB in patients undergoing maintenance HD or CRRT to those who did not receive RRT. Consistent with previous reports [16–20], daily and cumulative dose, treatment duration, and dosing interval for L-AMB were not significantly different between patients receiving maintenance HD or CRRT and those that did not receive RRT. Except for three maintenance HD and one CRRT cases, none of the maintenance HD and CRRT cases required adjustment of the L-AMB dose of ≥ 0.5 mg/kg/day. Additionally, we did not observe any evident difference in the incidence of adverse drug reactions, although the number of included subjects was limited. According to these findings, L-AMB may be used for patients undergoing maintenance HD or CRRT without any adjustments of its dosing, duration, or interval from treatment initiation.

L-AMB is retained in circulation for a prolonged period due to its lower volume of distribution and reduced renal and biliary clearances [6, 15]. AMB, which is released from L-AMB, is highly bound to plasma proteins such as albumin and α1-acid glycoprotein, enabling the retention of biologically active unbound AMB at a low level in plasma [23]. Therefore, although L-AMB, which has a particle diameter of approximately 100 nm, may remain in systemic circulation without removal by the dialysis membrane, biologically active AMB in plasma might not be significantly affected.

Here, a nearly twofold higher mortality was observed in the CRRT group than in the non-RRT group. Furthermore, higher incidences of septic shock and disseminated intravascular coagulation were identified in patients undergoing CRRT. Patients undergoing CRRT were assumed to be critically ill, because the proportion of patients treated with catecholamine was higher for patients undergoing CRRT (17/19, 89%) than those that did not receive RRT (136/842, 16%) between the day before L-AMB treatment initiation and 7 days after treatment termination (*P* < 0.001). L-AMB was used as the first-line antifungal agent in most patients undergoing CRRT (12/19, 63%). In 19 CRRT patients, all seven patients who did not receive L-AMB as the first-line drug died; however, four patients treated with L-AMB as the first-line antifungal agent were discharged, in part, owing to improvements in their conditions. Of the 4 patients, two had transitioned to maintenance HD and two had discontinued RRT. Of the 8 deceased patients who were administered L-AMB as the first-line drug, five died within the day following the completion of L-AMB administration. Therefore, L-AMB might be empirically used in critically ill patients undergoing CRRT owing to its broad-spectrum fungicidal effect.

This study has several limitations. First, the generalizability of these findings should be carefully considered. The database employed herein did not contain data from university hospitals that may employ infectious disease experts or facilities with fewer than 200 beds. Furthermore, tracking transfers from or to other hospitals could not be conducted. Therefore, the results might not represent the daily practice of L-AMB treatment in Japan. Second, severity was not fully evaluated as data for APACHE II could not be obtained. Finally, the number of patients, especially those requiring evaluation of their incidence of adverse drug reactions, was low. Further studies with a larger number of cases from real-world databases and prospective studies are warranted to verify the results obtained in this study.

## Conclusion

In this study, we revealed that daily and cumulative dose, treatment duration, and dosing interval of L-AMB were not significantly different between patients undergoing maintenance HD or CRRT and those who did not receive RRT. Although the number of subjects was limited, there was no evident difference in the incidence of adverse drug reactions. Therefore, L-AMB may be used to treat patients undergoing maintenance HD or CRRT without any adjustments of its dosing, duration, or interval.

## References

[1] Kleinberg M (2005). Aspergillosis in the CLEAR outcomes trial: working toward a real-world clinical perspective. Med Mycol.

[2] Brown JM (2004). Fungal infections in bone marrow transplant patients. Curr Opin Infect Dis.

[3] Marr KA, Carter RA, Crippa F, Wald A, Corey L (2002). Epidemiology and outcome of mould infections in hematopoietic stem cell transplant recipients. Clin Infect Dis.

[4] Morgan J, Wannemuehler KA, Marr KA, Hadley S, Kontoyiannis DP, Walsh TJ, Fridkin SK, Pappas PG, Warnock DW (2005). Incidence of invasive aspergillosis following hematopoietic stem cell and solid organ transplantation: interim results of a prospective multicenter surveillance program. Med Mycol.

[5] O’Brien SN, Blijlevens NM, Mahfouz TH, Anaissie EJ (2003). Infections in patients with hematological cancer: recent developments. Hematology Am Soc Hematol Educ Prog.

[6] Stone NR, Bicanic T, Salim R, Hope W (2016). Liposomal Amphotericin B (AmBisome): a review of the pharmacokinetics, pharmacodynamics, clinical experience and future directions. Drugs.

[7] Laniado-Laborin R, Cabrales-Vargas MN (2009). Amphotericin B: side effects and toxicity. Rev Iberoam Micol.

[8] Barrett JP, Vardulaki KA, Conlon C, Cooke J, Daza-Ramirez P, Evans EGV, Hawkey PM, Herbrecht R, Marks DI, Moraleda JM, Park GR, Senn SJ, Viscoli C (2003). Amphotericin B Systematic Review Study Group. A systematic review of the antifungal effectiveness and tolerability of amphotericin B formulations. Clin Ther..

[9] Loo AS, Muhsin SA, Walsh TJ (2013). Toxicokinetic and mechanistic basis for the safety and tolerability of liposomal amphotericin B. Expert Opin Drug Saf.

[10] Kato H, Hagihara M, Yamagishi Y, Shibata Y, Kato Y, Furui T, Watanabe H, Asai N, Koizumi Y, Mikamo H (2018). The evaluation of frequency of nephrotoxicity caused by liposomal amphotericin B. J Infect Chemother.

[11] Álvarez-Lerma F, Soriano MC, Rodríguez M, Catalán M, Llorente AM, Vidart N, Garitacelaya M, Maraví E, Fernández E, Alvarado F, López M, Alvarez-Sánchez B, Espinosa J, Quintana E (2012). Study Group of Liposomal Amphotericin B in the ICU. Impact of liposomal amphotericin B on renal function in critically ill patients with renal function impairment. Rev Esp Quimioter..

[12] Penack O, Schwartz S, Martus P, Reinwald M, Schmidt-Hieber M, Thiel E, Blau IW (2006). Low-dose liposomal amphotericin B in the prevention of invasive fungal infections in patients with prolonged neutropenia: results from a randomized, single-center trial. Ann Oncol.

[13] Noguchi S, Takahashi N, Ito M, Teshima K, Yamashita T, Michishita Y, Ohyagi H, Shida S, Nagao T, Fujishima M, Ikeda S, Ito I, Fujishima N, Kameoka Y, Saitoh H, Tagawa H, Hirokawa M, Sawada K (2013). Safety and efficacy of low-dose liposomal amphotericin B as empirical antifungal therapy for patients with prolonged neutropenia. Int J Clin Oncol.

[14] Lee JW, Amantea MA, Francis PA, Navarro EE, Bacher J, Pizzo PA, Walsh TJ (1994). Pharmacokinetics and safety of a unilamellar liposomal formulation of amphotericin B (AmBisome) in rabbits. Antimicrob Agents Chemother.

[15] Bekersky I, Fielding RM, Dressler DE, Lee JW, Buell DN, Walsh TJ (2002). Pharmacokinetics, excretion, and mass balance of liposomal amphotericin B (AmBisome) and amphotericin B deoxycholate in humans. Antimicrob Agents Chemother.

[16] Goto K, Makino T, Ohchi Y, Shiromi R, Tsubone T, Abe T, Yamamoto S, Yasuda N, Hidaka S, Noguchi T (2012). Efficacy and safety evaluation of liposomal amphotericin B (L-AMB) for injection in patients with septic acute kidney injury during continuous renal replacement therapy (RRT). J New Rem Clin.

[17] Álvarez-Lerma F, Rodriguez M, Soriano MC, Catalán M, Llorente AM, Vidart N, Garitacelaya M, Maravi E, Fernández Rey E, Alvarado F, López-Sánchez M, Alvarez-Sánchez B, Granado D, Quintana E (2013). Study group of liposomal amphotericin B in the ICU. Effectiveness of liposomal amphotericin B in patients admitted to the ICU on renal replacement therapy. Rev Esp Quimioter..

[18] Bellmann R, Egger P, Gritsch W, Bellmann-Weiler R, Joannidis M, Kaneider N, Dunzendorfer S, Wiedermann CJ (2003). Amphotericin B lipid formulations in critically ill patients on continuous veno-venous haemofiltration. J Antimicrob Chemother.

[19] Kajiwara K, Tomita M, Okamura K, Nakagawa T, Sakanashi A, Miura R, Fujimoto K, Kajiwara N, Iwata Y, Nobuoka M, Matsunaga A, Yamamoto S, Oobayashi H, Yoshida K (2015). Pharmacokinetics of liposomal amphotericin B during hemodialysis using polysulfone/high-flux membrane. J Jpn Soc Dial Ther.

[20] Furukubo T, Tsuboniwa N, Katoh Y, Matsumoto K, Morita K, Matsunaga C, Maehara C, Izumi S, Shoji S, Yamakawa T (2012). Long-term monitoring of serum amphotericin B in a hemodialysis patient receiving a liposomal preparation for Cryptococcus infection. Jpn J Chemother.

[21] Hidaka S, Goto K, Hagiwara S, Iwasaka H, Noguchi T (2010). Doripenem pharmacokinetics in critically ill patients receiving continuous hemodiafiltration (CHDF). Yakugaku Zasshi.

[22] Matsumoto K, Ueno K, Ishida S, Kusumoto S, Mitsutake K (2003). Studies on therapeutic drug monitoring and evaluation of optimal dosage based on fluconazole pharmacokinetics. Jpn J Pharm Health Care Sci.

[23] Bekersky I, Fielding RM, Dressler DE, Lee JW, Buell DN, Walsh TJ (2002). Plasma protein binding of amphotericin B and pharmacokinetics of bound versus unbound amphotericin B after administration of intravenous liposomal amphotericin B (AmBisome) and amphotericin B deoxycholate. Antimicrob Agents Chemother.

