# Correction to: The clinical usage of liposomal amphotericin B in patients receiving renal replacement therapy in Japan: a nationwide observational study

**DOI:** 10.1007/s10157-020-02004-5

**Published:** 2021-01-08

**Authors:** Yoko Obata, Takahiro Takazono, Masato Tashiro, Yuki Ota, Tomotaro Wakamura, Akinori Takahashi, Kumiko Sato, Taiga Miyazaki, Tomoya Nishino, Koichi Izumikawa

**Affiliations:** 1 Department of Nephrology, Nagasaki University Hospital, 1-7-1 Sakamoto, Nagasaki, 852-8501 Japan; 2 Department of Infectious Diseases, Nagasaki University Graduate School of Biomedical Sciences, 1-7-1 Sakamoto, Nagasaki, 852-8501 Japan; 3 Department of Respiratory Medicine, Nagasaki University Hospital, 1-7-1 Sakamoto, Nagasaki, 852-8501 Japan; 4 Nagasaki University Infection Control and Education Center, Nagasaki University Hospital, 1-7-1 Sakamoto, Nagasaki, 852-8501 Japan; 5 Medical Affairs Division, Sumitomo Dainippon Pharma Co., Ltd, 1-13-1 Kyobashi, Chuo-ku, Tokyo, 104-8356 Japan; 6 Deloitte Tohmatsu Consulting LLC, Marunouchi Nijubashi Building, 3-2-3 Marunouchi, Chiyoda-ku, Tokyo, 100-8361 Japan

## Correction to: Clinical and Experimental Nephrology 10.1007/s10157-020-01989-3

The article “The clinical usage of liposomal amphotericin B in patients receiving renal replacement therapy in Japan: a nationwide observational study”, written by Yoko Obata · Takahiro Takazono · Masato Tashiro · Yuki Ota · Tomotaro Wakamura · Akinori Takahashi · Kumiko Sato · Taiga Miyazaki · Tomoya Nishino, and Koichi Izumikawa, was originally published electronically on the publisher’s Internet portal on 11th November 2020 without open access. With the author(s)’ decision to opt for Open Choice, the copyright of the article changed on 7th November 2020 to © The Author(s) 2020, and the article is forthwith distributed under a Creative Commons Attribution 4.0 International License (https://creativecommons.org/licenses/by/4.0/), which permits use, sharing, adaptation, distribution, and reproduction in any medium or format, as long as you give appropriate credit to the original author(s) and the source, provide a link to the Creative Commons license, and indicate if changes were made. The original article has been corrected.

